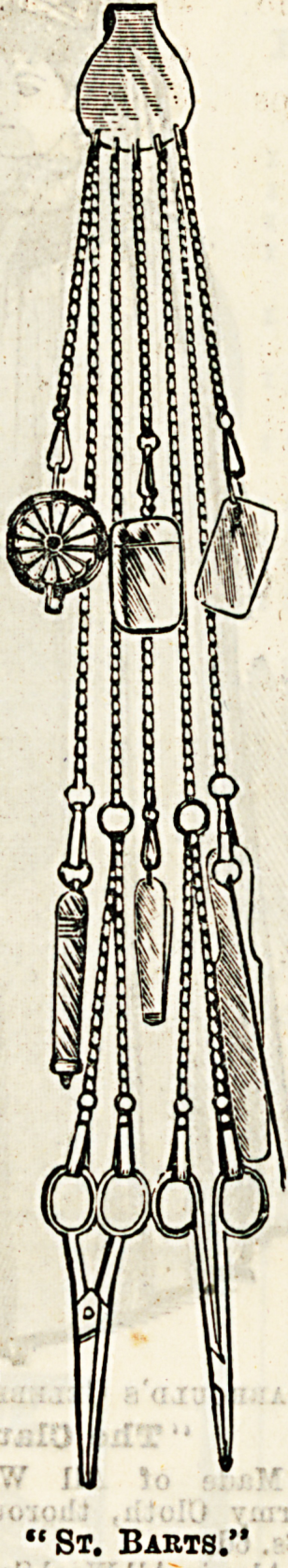# The Hospital Nursing Supplement

**Published:** 1895-02-09

**Authors:** 


### "The Hospital" Nursing Mirror.

Being the Extra Nursing Supplement of "The Hospital" Newspaper.

[Contributions for this Supplement should be addressed to the Editor, The Hospital, 428, Strand, London, W.C., and should have the word "Nursing" plainly written in left-hand top corner of the envelope.]

### News from the Nursing World.

### OUR PRINCESS.

The announcement of the return of H.R.H. the Princess of Wales, after her long absence from England, was most welcome to all our readers. At noon on Tuesday brilliant sunshine suddenly appeared, brightening even London streets, whilst stretches of untrodden snow in parks and gardens glistened dazzlingly. H.R.H. the Prince of Wales and the Princesses Victoria and Maud came up from Sandringham to Marlborough House on Monday, and the Duke and Duchess of York also arrived in town, whilst the Marquis and Marchioness of Bute were waiting on the platform to welcome "Our Princess" at Charing Cross. We may well add our own cordial congratulations on the safe return of Her Royal Highness. She is dear to each one of her subjects, but more especially to the nurses with whom as President of the Royal National Fund she has particularly identified herself.

### THE COLLECTING BOX.

A variety of opinions were expressed at the meeting held on Monday at the Hotel Victoria to consider "Street Begging for Charities by Children." When a youngster suddenly shakes a collecting box in front of the pedestrian, demanding a donation towards some hospital, the title of which is scrawled in illiterate fashion on an odd piece of paper, most people take leave to doubt the ultimate destination of the coins. These children, of both sexes, generally dart forth unexpectedly from dark corners, and are apt alike to resent questions or denials. The smaller and prettier the child the more likely is it to be successful in its appeals to those who would rather "spare a penny" for an unworthy object than take the trouble to investigate the claims of other charities, to which they, perhaps, could well afford a handsome donation. Whilst a judicious home training aims at teaching the little ones that they cannot have everything they ask for, experience in street begging proves to them that reiterated demands meet with comparative success; it is, therefore, hardly possible that youngsters should fail to deteriorate under such conditions, whether they beg for worthy or unworthy objects.

### COLNEY HATCH ASYLUM.

The fresh and healthy appearance of the Colney Hatch Asylum attendants is a visible proof of the beneficial effects of regular exercise taken in good air. Daily walks form an important feature in the treatment of many mental cases, and when the weather is reasonably good it is the custom for patients to spend about four hours daily in the open air, attended, of course, by their nurses. The uniform worn by the latter consists of a dark blue dress with red collar and waistband, and white linen aprons with square bibs, the charge nurses being distinguished by turned down linen collars and a different waistband, while particularly neat and becoming caps are worn by all. A large recreation room is provided for the nursing staff, who sleep in small single cubicles. Lectures on First Aid are given by one of the doctors, and examinations at the completion of each course are held by the medical superintendent of some other asylum. A pleasant air of comfort and general kindliness surrounds the workers at this great county asylum.

### LEWISHAM INFIRMARY.

The Local Government Board in making known to the Lewisham Guardians the results of the "inquiry" held at the infirmary, suggests that an opportunity be given for the medical superintendent and matron to carry out their respective duties during the coming six months, and it is much to be hoped that both these officers will in future sink individual differences and work together for the sick poor for whose treatment and nursing they are severally responsible.

### THE CENTRAL SICK ASYLUM.

There is so little to commend in the structure of this infirmary that it is pleasant to find other things to praise, one of the most observable being the cheerfulness with which the nursing staff make the best of such inconveniences as are irremediable. However, the long-needed bath-room for their exclusive accommodation is at present under consideration, and its erection may be anticipated in the near future. Improvements in the dormitories, although desirable, hardly seem at present practicable, but it is to be hoped that the probationers, for whom the medical superintendent and the matron do so much educationally, may by and by be more suitably housed. The patients are well clothed, fed, and nursed, and the old-fashioned wards are clean and orderly, and would be greatly brightened up by some good plants and flowers. Although a few of these are supplied by the nurses' liberality, there is room for many more, which might be offered by visitors who realise the depressing effects of monotonous and unlovely surroundings. Palms and indiarubber plants live for years in wards, repaying a hundredfold in pleasure the pains bestowed on them. We venture to think that anyone bearing one of these to the Central Sick Asylum, Cleveland Street, W.C., would receive a most courteous welcome from Miss Elma Smith, the matron.

### A NURSE FOR KNOTTINGLEY.

A meeting was recently held at the Town Hall, Knottingley, to discuss the institution of a trained district nurse. Miss A. Hughes, Superintendent of the Central Training Home for the Queen's Jubilee Institute, made an excellent speech, which was followed by an eloquent address by Lady Ramsden, who, from thirty years' personal experience of Yorkshire men and women, expressed her confidence in the kindly co-operation which might be reckoned upon from the working classes in the present scheme.

### ANDOVER COTTAGE HOSPITAL.

This pretty cottage hospital, which a generous donor gave to the town, contains eight beds for non-infectious cases. Patients of both sexes are admissible from all parishes situated within the Andover Union. The hospital appears to be appreciated and to fill an obvious want, as those of Salisbury and Winchester are too distant to be available for local accidents or urgent illnesses. Although all patients pay, according to their means, towards their maintenance, and the lady superintendent receives no salary, the financial position of the hospital cannot be considered satisfactory, as expenditure exceeds income, and the subscription list needs enlarging, and so does the stock of linen, &c. The Samaritan Fund managed by a ladies committee was organised last year, and has already done good work. Fresh interest seemed awakened by the public meeting on the 30th ult., at which the eighteenth annual report was presented, various schemes for adding to the funds during the current year being advocated by the indefatigable superintendent, Miss Botham, and the committee. In the evening a successful soirée took place, at which a large number of guests were entertained by the Mayor and Mayoress. The programme consisted of vocal and instrumental music, and an original hospital story recited by Miss Gethen (formerly Sister Queen). The Mayor, in the course of a short speech, wished success to the Cottage Hospital, the funds of which ought to considerably benefit by the proceeds of this well-attended social gathering.

### ARE ADVERTISEMENTS TABOOED?

"I feel like a conspirator about to take part in some mysterious rite," exclaimed a lady, who, after nearly an hour's wandering, accidentally discovered the place where a course of lectures on nursing was being given. When discovered it was found to be situated at the end of a narrow passage, there was no light without or within the bare schoolroom, nor did any printed bill indicate that a course of talks on sick nursing was in progress, and yet it was a public course under the management of the County Council. Inquiries of shopkeepers had proved useless, for there were no public notices; even the local clergy had never heard of the instruction provided free in their own parishes. Needless to say the audience was accommodated easily on a single bench, and the trained nurse—an accomplished lecturer—had the privilege of addressing half-a-dozen people in a chilly room, whilst scores of working women within a stone's throw of the spot remained ignorant of a beneficent project for their instruction. In country centres preliminary printed notices are conspicuous, and local secretaries are energetic in securing fair audiences; but in the outskirts of the metropolis the most unattractive spots seem sometimes to be chosen, whilst no steps whatever are taken to ensure the attendance of those for whose benefit the County Council's educational advantages are nominally provided.

### THE TEMPERATURE OF BATHS.

The nurse at Wirral who accidentally scalded a child has been declared not guilty of the charge of manslaughter, and the verdict was received by those present in a manner which showed that the nurse's previous kind treatment of patients weighed with them. But the habit of testing the temperature of baths should so grow into a nurse that she should do it automatically, for if properly-trained she would be as little likely to put a patient into untried water as into an empty bath. It is to be feared that nurses like other people do not always carry into practice what they are taught, but one who does not habitually and carefully try the temperature of every bath she gives, may any day find herself on trial for manslaughter, and even if acquitted will be haunted for life with the terrible thought of having caused the death of her patient.

### METROPOLITAN OR PROVINCIAL?

An amusing story is told of a young lady who, after undergoing a limited period of training in a metropolitan hospital, offered herself as ward sister in a large provincial infirmary. "Of course I am not up to taking such a post in London, but I imagine you will be glad of me here," said the ingenuous applicant. The matron promptly took her to see her wards, listening in amused silence to the ejaculations provoked by the sight of novel appliances and methods in common use. "We had nothing of this kind at our hospital," the visitor remarked presently, "yet you say they are a year or two old here. I beg your pardon for my presumptuous application." "It is granted," said the Matron of the infirmary, "and I am glad my nurses did not hear your offer. Let me tell you that we have enforced the three years' training for about twenty years, and we don't feel that we have much need to take either lessons or nurses from metropolitan hospitals."

### QUEEN'S NURSES IN SCOTLAND.

The Scottish Branch of Queen Victoria's Jubilee Institute has issued its annual report. H.R.H. Princess Louise, President of the Council, takes much interest in the growth of the work, and Miss Guthrie Wright is the indefatigable hon. secretary. In the rules concerning the engagement of Queen's nurses for Scotland it is stated that the minimum training is two years in a general hospital approved by the Council, supplemented by six months in the District Training Home, attendance on courses of lectures and training in monthly nursing. For superintendents and for nurses going to country districts, three months in a maternity hospital is also requisite. The training of nurses for the sick poor is now commendably thorough, and the Queen's nurses enjoy well-deserved appreciation. Further particulars can be obtained from the hon. secretary or from the superintendent at 29, Castle Terrace, Edinburgh. The lectures to nurses on hygiene are given by Dr. Littlejohn; obstetrics, by Dr. Milne Murray and Dr Haig Ferguson; and on fevers, by Dr. John Thomson. Eight new branches have engaged Queen's nurses and become affiliated to the Institute during the year, and the Aberdeen and Hamilton Committees have been enabled by local support to purchase larger homes.

### SHORT ITEMS.

The Westhampnett Guardians have asked the Ladies' Committee to draw up a scheme for introducing training nursing into their Workhouse, and to report to the Board.—Sister Helen, of the Community of St. Lawrence, Belper, successfully passed the January examination of the London Obstetrical Society.—At a recent meeting of the East Ashford District Council a proposal was made to secure the services of two trained nurses for the sick poor in their own homes.—At the Leeds Workhouse Infirmary it is intended to do away entirely with pauper helpers, and to have a staff of six charge, fifteen ward nurses, and nineteen probationers.—The nurse at the Basing Union has asked for an inquiry into the nursing arrangements, and a committee of seven governors has been appointed to investigate matters.

### Elementary Anatomy and Surgery for Nurses.

By W. McADAM ECCLES, M.B., B.S., F.R.C.S., Lecturer to Nurses, West London Hospital.

### V.—THE PROCESS OF INFLAMMATION, AND THE HEALING OF WOUNDS.

IN these days the art of surgery, with the principles of medicine, is advancing rapidly, and both branches of the medical science owe no small amount of their progress to the sister profession of nursing. It is, or at any rate should be, the desire of physicians or surgeons and nurses to mutually help one another towards the goal of perfection, but this cannot be obtained unless each possess a thorough fundamental knowledge of the methods whereby nature performs her work of healing, and of the enemies she has to combat.

Both the surgeon and the nurse have to deal with a highly complex living organism, whose tissues stoutly resist any untoward interference, and will scarcely brook any maltreatment by the unlearned. A part exposed to violence will either be killed outright or it will recover its normal condition. The process by which the latter happy result ensues is by common consent termed inflammation. This may be defined as a series of changes which occurs in a living tissue, consequent upon an injury which is not in itself sufficient to cause death of the part. Inflammation, as its name implies, is allied to a process of combustion, and, like fire, it may be man's best friend or his worst enemy. When it proceeds up to a certain point, healing is the result; if it transgresses this line, a greater or less mischief will follow. It is very essential, therefore, to understand the chief points in this process.

Inflammation is evidenced by certain well-marked signs or symptoms, which have been observed accurately for ages. They are five in number, viz.: Redness, swelling, heat, pain, partial or complete loss of function of the part. These may be reviewed *seriatim*.

Redness: Any exposed area of skin over an acutely inflamed part will be found to be red owing to an excess of blood in the dilated vessels. Pressure of the finger in this region will produce a temporary blanching, consequent upon the small blood channels being emptied, but quickly refilling when the pressure is removed.

Swelling is produced in part by the larger amount of blood in the inflamed region and partly by the fluid and blood cells which have passed from the vessels into the tissues around.

Heat is the outcome of the excess of blood and the increased vital activity of the tissue itself.

Pain is in great measure due to the tension caused by the swollen condition of the part.

Interference with function is readily seen and appreciated. The organ which is inflamed is thrown, so to speak, out of gear, and its work will be either badly done or not performed at all.

Certain technical terms are frequently employed when the process of inflammation is under consideration. The word congestion implies an early stage of inflammation, and designates a fulness of the blood vessels and thus of the tissues. It is of two varieties—active when there is active dilatation of the blood vessels, and passive when the increase in the amount of blood is due to an interference with the proper return of venous blood. The former is daily evidenced in a typical manner by the true inflammatory redness; the latter in the tissues of a limb, the seat of varicose veins. This congestion of a part leads to the escape of the contents of the blood vessels into the parts which surround them, thus producing an effusion into the tissues. This effusion may later be absorbed into the veins or lymphatics.

The inflammatory process may terminate in one of four different ways: Resolution, suppuration, becoming chronic, and gangrene. It is necessary to define these further. Resolution occurs when the inflamed part returns to its normal condition without the formation of pus or matter. Suppuration, or the formation of pus, is by no means an infrequent termination of an inflammatory process. Pus is a thick fluid composed essentially of a liquid portion, and in this a number of solid particles known as pus cells. These corpuscles or cells are derived chiefly from the white corpuscles of the blood which having migrated into the tissues from the blood vessels have there died and partly degenerated. They are probably, however, partly derived from the inflamed tissues themselves. It is now almost unanimously agreed that should pus form either in the tissues as an abscess, or on the surface as in ulceration, it is caused by the action of parasitic micro-organisms known as bacteria. In wounds made by the surgeon it is of the utmost importance to avoid any infection by these micro-organisms, so that there may be no formation of pus.

When suppuration occurs the bodily temperature is raised, fever is present, and not infrequently there is a temporary sensation of chilliness or an actual fit of shivering, which is known as rigor. In the next lecture the subject of the prevention of suppuration will be dealt with. If inflammation become chronic, a great deal of scar tissue will be formed, and this has a tendency to contract. This contraction may subsequently lead to serious consequences, such, for instance, as the narrowing of a tube or channel of the body, leading to a stricture of the same. Sometimes the inflammatory process is so intense that the blood supply of the part is cut off chiefly by the pressure of the inflammatory products. The direct result of this in certain cases is death of the part, or gangrene. In such a case the dead portion has to be separated from the living at the expense of the latter by a process of ulceration.

Ulceration, or molecular death, *i.e.*, death of a portion of tissue bit by bit, is in contradistinction to sloughing, where an area dies *en masse*. The core of a boil, or the central part of a carbuncle are good examples of sloughing caused by intense inflammation.

Wounds heal practically by one of two ways—without the formation of pus or with suppuration. The former is the ideal method, and its production will occupy our attention in the next lecture.

### Addenbrooke's Hospital, Cambridge.

WE are happy to be able to state that the Court of Governors of this hospital, at their meeting on Monday, the 4th inst., unanimously adopted the whole of the recommendations of the committee, as embodied in the printed report, the details of which were given in THE HOSPITAL of January 12th. Mr. Wood and his supporters did not attend the meeting, and it is fair to conclude that they felt the position they took up was untenable. The committee have been reappointed with power to consider the best way of carrying the recommendations into effect. Mr. Burdett, Mr. Keith D. Young, and Mr. Fawcett, the architect, are to prepare a report embodying their views, and we understand there is no doubt that they will be promptly acted upon. As showing the interest which has been excited in Cambridge and the neighbourhood by the report of the committee, we are glad to be able to state that Mrs. Towry Hall has sent a cheque for £1,000 to the funds, making, with the gift of the treasurer, £2,000 in special donations towards the work, so that the committee will be fully justified in placing the accommodation for nurses at Addenbrooke's on so modern and complete a footing that it will raise the institution at once to the position of prominence and importance which it ought long ago to have occupied. We look forward to the time when the nurses' training school connected with Addenbrooke's Hospital, Cambridge, will be so efficient and popular that every nurse graduate who obtains its certificate will be recognised in the field of nursing as bearing credentials as valuable and pre-eminent as are those which at present attach to the degrees of Cambridge University in the profession of medicine.

### Orphans Still Caged.

Is it the recollection of the large sums of money spent on the erection of the dormitory "cages" at Kilburn which makes the Church Extension Association so unwilling to part with them? Public attention having been called by The Hospital to the dangers of a system which confines helpless children in iron cubicles secured from the outside, certain alterations have taken place. In some cases the little folks can now open their own doors and in doing so set an electric bell ringing in the room of the sister in charge, adjoining the ward. Although this innovation has obvious advantages, the method leaves much to be desired, and many elements of danger remain. But the bolts and bars have only been replaced by bells to a limited extent. The change was made after the appearance of the accompanying illustration in The Hospital of Dec. 1, 1894, and we learn that there is no intention on the part of the authorities to alter the fastenings of the cubicles of the smaller children, of whom some forty are confined in one dormitory by means of four separate bolts. These are at the end next the fire place, and farthest from the doorway. In any emergency of fire or other accident these forty little creatures therefore cannot be liberated until all four bolts are opened by a (probably) panic-stricken woman, and it is not unlikely that under such circumstances the process may be beset with difficulties. It seems that nothing less than an official "inquiry" will suffice to protect the babies from kindly-intentioned but ill-advised treatment. With the constant appeals of the Church Extension Association every one is familiar, and contributors certainly share equally with the recipients the responsibility of seeing that funds are properly expended. If an annual balance-sheet reached the subscribers they would doubtless have realised ere this that such "cages" are costly as well as dangerous investments. All who see them must agree that no consideration of wasted money should interfere with the immediate banishment of the entire doors or, better still, of the cages themselves. The introduction of such an un-English method into any orphanage is deeply to be deplored. Surely, in addition to the food and clothing and instruction meted out so liberally, the good "sisters" can add a moral training, which needs no emphasis from bolts and bars!

### Notes From Cairo.

What more exhilirating thought for the invalid than that of escaping the English winter, its cold, fogs, short days, and the inevitable indoor life entailed by these.

What land more attractive than Egypt, with its dry, warm climate and endless sunshine, the certainty that December and January may be spent out of doors in cotton dresses with large sunshades and iced drinks. The journey from London to Cairo only takes six days, and on no route is the traveller more considered.

Cairo is a city of magic beauty, and it is difficult to realise that precautions for health are necessary in this lovely climate. But the delicate must at once learn the conditions necessary to well being. Woollen under-garments are needed by day and night, and the windows must always be closed during the night. A warm wrap is necessary at sunset —that dangerous but fascinating hour! The invalids then hasten home, forbidden by their doctors to watch the sunset glories from desert or citadel, for the mists that add a beauty to the land of colour are unhealthy. Drinking-water must be chosen with care, and the wisest plan is to drink aerated waters or light wines. The native bazaars, so tempting to the stranger, are sources of infection to Europeans. They are in the most crowded part of Cairo, which is quite undrained. It is wise to rest indoors from twelve to three; the sun is very hot then, and there is great risk of taking cold on returning in a heated condition to the cool interior of the hotel.

Cairo affords excellent medical attendance and nursing. The English doctors are experienced and kind, and one of them is the head of the home for English nurses. Nurses can be secured at the hotel, where every consideration is shown in illness, and complete apparatus is kept for invalid cookery. The noise of hotel life is a drawback, but this depends chiefly on the situation of rooms or on one's next door neighbours. It is an expensive way of being nursed, as, besides personal hotel expenses, there are those of the nurse, about 15s. a day and her fee of from three guineas a week, besides the doctor and medicine.

There is an English nursing home with its English matron and nurses where excellent food is provided, and milk comes from a special farm and the water is bottled at Malvern. The house, which was once a haarem in the Khedive's family, is in a quiet, pleasant situation, and has a nice garden. The bed-rooms are comfortable and pleasant, and each patient has one to himself. When convalescent he can go to table d'hôte. Visitors are allowed at the doctor's discretion, and in some cases a friend can accompany the patient, paying at the same rate. The fees are a guinea a day, not including doctor, medicines, or stimulants.

The German Hospital is nursed by deaconesses and is in charge of a German doctor; all speak English and have many English patients. The hospital is in a good situation, and the patients do well there. A private room is 10s. a-day, sharing a room with one other patient 5s. a-day, and the general ward is a still lower figure. All the charges include medical attendance and medicine.

No infectious cases are kept in hotels or hospitals, but are taken on board a dahabeah on the Nile, of which one or two nurses are in charge. It is a comfortable house-boat, and contains saloon, bed-rooms, and bath-rooms.

The native hospital and medical school are in charge of English doctors, and though in the Kasr-el-aini the wards are under the care of English sisters, they are assisted by arabs, and the accommodation and food are unsuited to Europeans.

### Nursing in the West Indies.

By a Nurse Who Has Been There.

A little information about these beautiful islands may be of interest to my professional sisters who read in The Hospital of the need for trained nurses there.

The small but beautiful island of Barbados, called Little England, is perhaps actually more unlike our native land than any other spot, yet it is dear to British subjects who cling to British ways.

A nurse can go out by the Scrutton line of steamships (generally starting from the south West India Dock) at a much lower cost than by the Royal Mail, the former only charging £17 10s. first-class fare, and reaching Barbados in from sixteen to twenty-one days. I saw the island first in the month of December after a delightful passage of sixteen days. It lies low, and consequently is not visible till one is within an hour and a half's steam of it. It comes as a sudden vision of loveliness. Entering the bay the tropical sun shines gloriously, reflecting its glorious rays in every ripple of the deep blue waters. Above the shell-strewn beach the feathery palms and grand tamarind trees rise, and rest the eyes, which have looked on sea and sky incessantly during the voyage. When I landed and saw the scarlet Hibiscus flowers, flowering vines, the Eucharis lilies, and stephanotis in the gardens, I could not help comparing the scene with the dear old England wrapt in fog and frost.

A nurse intending to work "for herself" in Barbados needs good health, and she must not object to live in a kind of Turkish bath atmosphere, the general temperature being from 85 to 95 degs. Fahr., some days much hotter. There is a fresh breeze in the evening, and out at Hastings or Bathsheba the air is delightful. There is little English fruit or vegetables, except apples, now and then from the ice-house, a kind of general store. One cabbage costs a shilling, but a quarter of one can be bought; butter is about half-a-crown a pound, and a kind of grease called butter is sold in tins, but I should prefer bread dry. Milk is dear and scarce, owing to want of sufficient pasture for cattle, the island being so small. Meat also is scarce, and not very nice-flavoured. The chief vegetables are yams (sweet potatoes), eldoras (pumpkins), a kind of bean, and peas that are not very nice, tomatoes, a kind of spinach, and a few others. Bananas, chadocks, golden apples, maming apples, Pappau fruit (used when green in stews, when ripe as a fruit), custard apples, sour tops, star apples, semitos (passion flower fruit), a berry called plums, and others which pass for gooseberries and cherries, but are not one bit like our own. A pear, as large as a man's head, with a great stone inside, lapadillos, and mangos. Then one has to get used to mosquitoes, and to be well bitten for the first two or three years, in spite of a protecting net at night. The nightly concert of whistling frogs is awful till you grow callous to it, and to flying ants.

As regards clothing, thin white or light dresses, six at least, are necessary, and very thin flannels, if any; thread stockings and gloves, light walking shoes or boots, which are expensive and not good on the island, and a pale gray cloak of silk-finished alpaca with bonnet to match. White umbrella and Brussels net or muslin caps and a good stock of cambric cloth underclothing is desirable, and it is best to have only a veil of gossamer of a thick quality to the bonnet to wear over the face, as this is a great comfort. Dark blue glasses are needed on first going out. No nurse should venture to go out who has not sufficient for many "a rainy day," for she must wait before she secures work regularly. I only know of two trained nurses, though there may be more. One is matron of the hospital, and the other is a Barbadian who had some training out there, came to England for more, and returned with the L.O.S. diploma, for which she studied at Queen Charlotte's Hospital. She is also certificated as a masseuse. One or two really good nurses would be highly valued in Barbados, for many officers' wives live there besides other English ladies who often need good nursing.

There is a large garrison on the island, and a beautifully situated hotel. Barbados is the health resort of the West Indies, and I think two nurses trained for general work ought to succeed. In addition to their three years' certificate, they should be monthly nurses, if not midwives, and should understand massage treatment, and learn all they can about dysentary, cholera, and yellow fever, before going out. Many nurses are eager to have charge of those complaints and yet know little about them. Tropical nursing is different in some ways to that required in a temperate zone.

Medicine is very expensive in the West Indies, the writer has given one shilling for three Cascara Sagrada pills, so a nurse going out should take some bottles of capsules with her, also Eno's Fruit Salt, glycerine and cucumber for the skin, and some bottles of Scrub's Cloudy Ammonia for mosquito bites and the bath, also vaseline, boracic acid powder, and permanganate of potash, and plenty of good soap, and all requisites for plain or fancy work. Barbados leg is a complaint which nurses may never get themselves, but it is well to remember that it is not advisable to use cold water immediately after hot, in the tropics, or very bad arms may result, with inflamed lymphatics. The custom is to lay cloths, steeped in good spirit, over the part affected. Rum is oftenest used, and quinine is given internally. It is best to avoid alcohol in the tropics, unless ordered by a doctor.

Yellow fever is very infrequent at Barbados, but there are other parts of the West Indies where it is not so uncommon. The people of Barbados are kindness and good nature personified, and the coloured population simple and nice in the extreme.

All that is said of the want of trained nurses in Barbados applies equally to Demerara, in British Guiana, situated to the north of South America. It is three days steam from Barbados and not nearly so healthy, being surrounded by mud on the coast. The only water to drink is rain water, which is not unhealthy if filtered; the rainfall is tremendous and the place flat and damp, but George Town is a large and busy place, and much good service could be given to the doctors there, who would doubtless welcome skilled attendance. The Portuguese form a great portion of the population of George Town, and are staunch Catholics, having a magnificent cathedral and a fine church there, but I fear their ideas of nursing differ greatly from English ones.

There is a leper hospital up in the country, which contained 400 patients last summer, and there is a large infirmary for general diseases. The coloured people are somewhat unruly patients, and prefer to be nursed by their own people. Private nurses would probably be considered an acquisition by English residents in Demerara.

### Presentations.

Miss Bermingham, on resigning the matronship of the Croydon General Hospital, was presented by the nurses with a case of silver spoons, sugar-tongs, and initialed serviette ring. The case bore this inscription: "Miss Bermingham. A token of Affection from her Nurses. Croydon General Hospital, January 30th, 1895."

### Notes and Queries.

**Queries.**

(74) *Milk.*—Can you give me particulars as to the relative advantages of cow's and condensed milk for infants?—*Mater.*

(75) *Probationer.*—Where can I find names of children's hospitals? Can girls be admitted for training at 18?—*A. G.*

**Answers.**

(74) *Milk (Mater).*—You cannot do better than read a book just issued by the Scientific Press, entitled, "Infants' Feeding by Artificial Means," price 5s.

(75) *Probationer (A. G.).*—See Burdett's Annual, published by the Scientific Press. We do not think you would be taken at any hospital yet, you are too young.

### The Book World for Women and Nurses.

[We invite Correspondence, Criticism, Enquiries, and Notes on Books likely to interest Women and Nurses. Address, Editor, The Hospital (Nurses' Book World), 428, Strand, W.C.]

A Short Memoir of Emily Minet. Edited by the Rev. C. G. Gipp. (Rivington, Percival, and Co. Price 2s.)

The subject of this short biography must have been a remarkable woman, combining rare force and sweetness of character, and had she been willing to come forward, would no doubt have taken a leading position among the women-workers of her day. As it was, however, she preferred a quiet unostentatious sphere, doing excellent work and making herself beloved by all who came in contact with her. Miss Emily Minet took up an independent line early, becoming a governess at seventeen, even then showing in which direction her tastes lay by her interest in district visiting and nursing, and the active part she took during a serious outbreak of malignant diphtheria in the village where she was living. A few years later Miss Minet went to Barrow-in-Furness to start a middle-class school and to help at a cottage hospital which was built there in 1866. Four years later we find her at Walsall to be trained under "Sister Dora," after which she went to the Middlesex Hospital in London as Superintendent of Night Nurses; but the strain of night work was too much for her, and she was obliged to give it up after a few months, "greatly disappointed that owing to the shortness of her residence in the hospital the authorities could not grant her a nurse's certificate." The next post she filled was as head of the Home of Mercy at Llandaff, where, however, she only stayed a few months, resigning it to take up, in 1871, what was to be her chief work, as Lady Superintendent of the Nursing Home at Stratford-on-Avon, which was to supply nurses for the poor and also to train nurses. It was whilst thus engaged that Miss Minet's health showed signs of breaking down, and we cannot but regret that extra help was not granted her to relieve her of some of the arduous work and spare her longer to exercise the great personal influence that she possessed in so large a degree. She had a staff of eleven or twelve nurses under her, with whom she kept up a regular correspondence, and knew as "much about them when they were away from the Home on duty as when they were under her own eyes," and at a time when her duties were so exacting that she had "scarcely any time that she could call her own." Even during her short absences she was not free from the cares of this Home, but "twice a week regularly, at least, came letters with detailed instructions as to work, &c." In 1891 she had to give up her work, and died in August, 1892. It is very much to be regretted that so little material should be forthcoming for a full biography of Miss Minet, especially as regards her life at Walsall with Sister Dora.

Infant Feeding by Artificial Means: A Scientific and Practical Treatise on the Dietetics of Infancy. By S. H. Sadler. (Published by The Scientific Press, 428, Strand. Price 5s.)

Twenty-four illustrations and 234 pages of well-printed letterpress add to the attraction of this well-bound little volume. Mrs. Sadler does not pretend to originality, but rather to editing a patient and laborious collection of a vast number of facts and opinions from well-known authorities on the dietetics of infancy. Numerous quotations are given from the works of Dr. Cheadle, Dr. Starr, Dr. Goodheart, Dr. West, Dr. Playfair, Sir William Roberts, Dr. Pavy, Dr. Eustace Smith, Dr. Angel Money, Dr Parkes, Dr. Wynter Blyth, Dr. Routh, and others, including several foreign physicians. The book is in effect an exhaustive treatise on the various methods of hand-feeding, and should be welcomed as a most valuable work of reference by all persons interested in a matter which is sooner or later brought before the notice of most of us. An analysis of prepared foods, many useful recipes, and an index are also included in this instructive volume, and the evils of improper and the advantages of proper dieting are given on the authority of the eminent writers whose names we have mentioned. The illustrations show various methods of feeding, and also the sources from which some of the nourishments recommended is derived.

A new elementary text-book of anatomy by Dr. Henry E. Clark, editor of "Wilson's Student's Vade Mecum," will be published in about ten days by Messrs. Blackie and Son (Limited), Glasgow. Dr. Clark has written the book specially for the use of nurses in hospitals, and it contains a sufficient course of anatomy for those who merely require an elementary knowledge of the subject. It is profusely illustrated.

### Everybody's Opinion.

[Correspondence on all subjects is invited, but we cannot in any way be responsible for the opinions expressed by our correspondents. No communications can be entertained if the name and address of the correspondent is not given, or unless one side of the paper only be written on.]

### INGS HOUSE NURSES' CO-OPERATION.

M. L. Canning writes: The enclosed has been sent to me by this morning's post, and I write simply to say that in the event of your further inserting letters that may or may not be detrimental to this institution the solicitor has been instructed to take legal proceedings against you. Nurses who are worth having, or worth keeping, have not only a good home here, but also, as a rule, plenty of work. Therefore, before inserting any more libellous letters, I should strongly advise you to make some inquiries respecting the writer of them. I consider that you should uphold institutions, and assist matrons and superintendents in weeding out the many black sheep there are in the profession. Perhaps if you occupied the position of matron or superintendent for one week, you would be at any rate a little more particular. I have been one of The Hospital's supporters from its first edition, or rather number. As you have no true knowledge of this institution, good or bad, be kind enough to leave it entirely outside your editorial judgment.

[The enclosure referred to is a cutting from "Everybody's Opinion," headed "Spurious Co-operations and Nursing Homes," which appeared in our issue of January 26th, and we have no knowledge as to what institution our correspondent, the "Private Nurse," referred to.—Ed. *T. H.*]

### Begging Letter Writers.

From time to time we receive applications for help of various descriptions. These applications are carefully investigated, and in most cases we have found the objects of them both meritorious and needy, and it has been a great pleasure to us to afford most material assistance with the help of our readers. Recently a case has come before us from an applicant who may be distinctly classed with the habitual begging letter writing classes, and we have been consequently put to useless trouble in investigating the matter. We give publicity to this because we wish it to act as a warning in other instances of the kind, as in future we intend to make an example of persons who endeavour to abuse our power of affording them assistance. The case we refer to was one of a young man who was already provided for in an incurable home, and appeared to have ample means at command.

### Where to Go.

Westminster Town Hall.—A ball in aid of the Royal Free Hospital, Gray's Inn Road, will take place on February 20th. Tickets, 12s. 6d., of Mrs. Layland-Barratt, 39, Lennox Gardens, S.W., Secretary and Treasurer of the Ball Committee.

### Dress and Uniforms.

BY A MATRON AND SUPERINTENDENT OF NURSES.

### SOME USEFUL WALLETS.

Mr. W. K. Stacey, cutler and instrument maker (4, Newgate Street), is showing a large and well-varied assortment of wallets and chatelaines for nurses. These goods are con-

THE "LONDON." THE "DORA," (shewn open.) "ST. BARTS."

spicuous not only for usefulness and finish, but for the very moderate price at which they are offered to the purchaser. The "Dora" is a particularly useful wallet, and one that is sure to be popular, as, in addition to containing all the necessary instruments, it possesses the advantage of a flap which covers them in, thus favouring their immunity from dust and dirt. This wallet is made of grained calf leather, in black, dark blue, or brown, and is fastened by a spring catch, which is suspended by nickel-plated chains and hook from the waistband. The "Chelsea" and the "Dufferin," though on a rather different principle, are very compact and durable, and are made in black cowhide, which may be said to last for ever. In the "London" we see a chatelaine and wallet combined. It is perfectly noiseless, being made throughout of solid leather. It is complete in every way, and, as shown by the illustration, contains a large number of instruments. For those who remain faithful to the chatelaine there is the "St. Bart's," made in steel or nickel-plate, with eight chains to hold eight articles. We well remember the flutter of pride when as a probationer we first donned this desirable and ornamental requisite, and can highly recommend it from experience. The pocket cases deserve a word in conclusion. They are made in the best morocco, completely fitted, and fold into a space small enough to enable them to be conveniently carried in the pocket. Another useful little novelty is the pulse glass, which is on the plan of an hour glass, the sands being so arranged that they run down in fifteen seconds.

### FANCY GOODS AT THE WHOLESALE SUPPLY COMPANY.

This enterprising firm, which has its headquarters at 46, Market Street, Manchester, has recently issued a very comprehensive catalogue of clocks, watches, jewellery, and plated goods. The watches, which are enclosed in cases either plain burnished with monogram, or elaborately chased, are all made by certified workmen, and for fineness of mechanism, combined with all modern improvements, have few, if any, rivals at the price. An excellent English gold lever watch is warranted for ten years, and a trial of fourteen days is allowed in each case, before completion of purchase. There is also a good assortment of medals, which can be had in gold or silver, struck from original designs, at about half the usual cost. Sleeve links and studs are other novelties calling forth admiration, as are also the clocks, some of which are especially suitable for institution use, being provided with a cathedral gong on which the hours and half-hours strike. A number of fascinating articles adapted for presentation purposes arrest the attention next, and our readers will be readily helped out of any dilemmas in the matter of choice in such matters, by sending for the catalogue of the Wholesale Supply Company.

Few establishments can compete with Messrs. T. Venables and Sons (Whitechapel Road) in the variety, quality, and cheapness of their wares. Their range is a very extensive one, and embraces the details of underclothing and those of furnishing. Though catering, as will thus be seen, for every section of the public, they pay special attention to nurses' uniforms. A large quantity of both regulation and fancy patterns are kept in stock, from which a selection can be made, and, if so required, the material made up by a competent staff of workers. The convenience of this arrangement is at once obvious, and will be appreciated by those of our readers whose spare time is limited. The favourite "Princess" shaped bonnet is kept in black, blue, grey, or brown straw, trimmed with velvet to match, at a most reasonable price. A gossamer veil the same colour is added, according to the taste of the wearer. Cambric strings are supplied with the bonnet, or they can be had separately at the cost of two or three pence, neatly made with a hem and narrow tucks. There are several shapes in cloaks; the plain circular, however, in black or navy blue Imperial cloth, drapes so becomingly to the figure that we must at once accord it the palm. The ordinary nurse's apron, with bibs and straps, is kept ready made in a nice quality of linen, and at a price so modest that we wonder how it is possible to make any profit out of it. A few words in conclusion must be devoted to the boot and shoe department. It is complete in every particular. The ward shoes are excellent in shape and finish, the necessary support for the instep is duly provided for in the shape of a strap, and the leather is both soft and durable. Felt slippers for night wear in various shapes, and hygienic boots will commend themselves both for their comfort and their neat appearance.

### Appointments.

THE VICTORIA INFIRMARY, NORTHWICH.—Miss Margaret Cole has been made Matron of this institution. She was trained at the Northern Hospital, Liverpool, and held the post of charge nurse at the Royal Albert Edward Infirmary, Wigan, and takes many good wishes to her new work.

HOME FOR INCURABLES, UPPER PARLIAMENT STREET, LIVERPOOL.—Miss Dorothy K. Ruston has been appointed Lady Superintendent of this Home. She was trained at the Royal Southern Hospital, Liverpool, and subsequently held the posts of night sister, sister of the children's ward, and for the last five years that of sister of the male surgical wards. We cordially congratulate Miss Ruston on her appointment. Her testimonials are excellent.

## Figures and Tables

**Figure f1:**
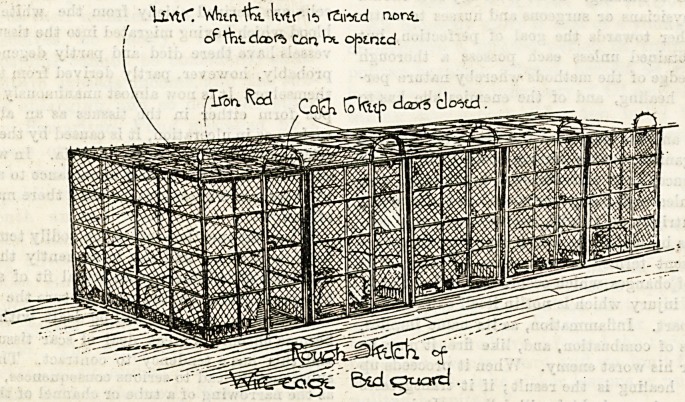


**Figure f2:**
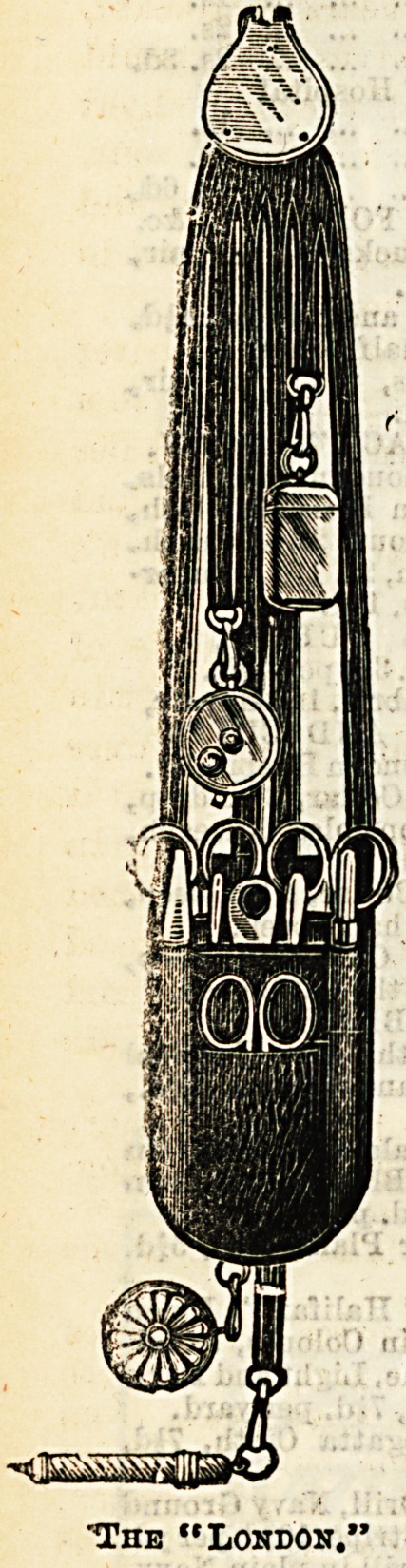


**Figure f3:**
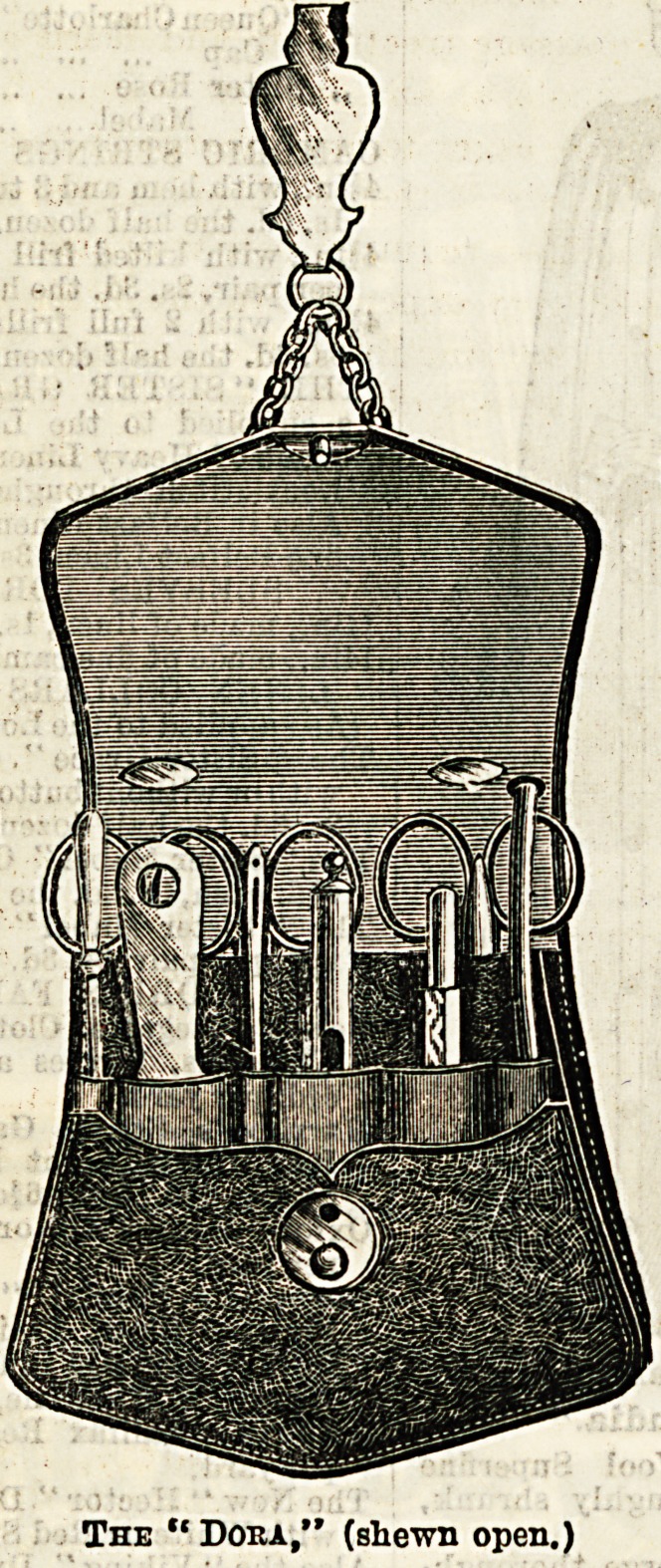


**Figure f4:**